# Managed pollination is a much better way of increasing productivity and essential oil content of dill seeds crop

**DOI:** 10.1038/s41598-022-17397-4

**Published:** 2022-07-30

**Authors:** Narottam Kumar Meena, Ram Swaroop Meena, Ravindra Singh, Arvind Kumar Verma, Sharda Choudhary, Balraj Singh, Ram Dayal Meena, Ravi Y, Murlidhar Meena

**Affiliations:** 1 ICAR-National Research Centre on Seed Spices, Ajmer, Rajasthan 305206 India; 2 All India Coordinated Research Project on Honeybees, ICAR-Indian Agricultural Research Institute, New Delhi, India

**Keywords:** Ecology, Plant sciences, Zoology

## Abstract

Dill seeds (*Anethum* *graveolens* L.) is the most valuable medicinal seed spice crop of Apiaceae. It bears small yellow flowers in the form of umbels. Being a cross-pollinated crop, floral visitors play vital role in pollination and seed sets. Hence, the present study was conducted at the ICAR-National Research Centre on Seed Spices, Ajmer (Rajasthan), India to discover the pollinator’s community, foraging behaviour and abundance of most frequent pollinators and different modes of pollination on seed yield and quality of this seed spice crop. The insect visitors community of dill seeds was composed of 28 insect species belonging to 14 families of 6 orders. Most of floral visitors started their foraging activity at 8.00 h, reached peak activity between 12.00 and 14.00 h and their activity ceased at 18.00 h. *Apis* *florea,* *A.* *dorsata,* *A.* *mellifera*, solitary bee, *Halictus* sp. and two unidentified species of Hymenoptera; *Episyrphus* *balteatus* (DeGeer), *Episyrphus* sp., *Eristalis* sp and two other *Musca* species of Diptera were identified as potential and regular floral visitors of dill seeds. The highest seed yield of 1505.63 kg/ha was recorded in the treated plots provided with only 10% jaggery solution and was at par with the open pollination. A lower seed yield of 1432.5 kg/ha was recorded in plots pollinated only with *A.* *mellifera* inside insect cages. Open pollination with 10% jaggery solution spray increased the seed yield of dill seed crop by 57%, one-thousand seed test weight by 96% and the essential oil content by 27% over control plots. These results show that managed pollination is a much better way to enhance yields and quality of dill seed crop than other treatments including only honeybee-based pollination.

## Introduction

Pollination is one of the most important ecosystem services to agriculture, once 75% of the food crops and nearly 90% of wild flowering plants rely on animal pollination, in different degrees^1,2^. Ollerton et al.^3^ reported that 87.5% flowering plant species are pollinated by animals, whereas, remaining plant species are either wind-pollinated or completely reliant on autonomous seed production^4,5^. Insect pollinators, particularly bee species visit flowers of angiosperm plants to collect nectar and pollens, thereby the visiting plants get pollinated and also enhance the production as well as quality. Non-availability of effective pollinators during flower anthesis leads to massive loss in the agricultural production. Gallai et al.^6^ reported annual value of pollination service to €153 billion in 2005, contributing 9.5% of agricultural food production crops. Further, the annual market value of additional crop production directly linked with pollination services is estimated at $235bn-$577bn worldwide^7^ In the absence of animal pollination, a potential annual net loss of economic welfare of $160 billion-$191 billion incurred globally^2^. In the last few years, there has been substantial increase in the value of this service due to decreasing numbers of insect pollinators, mainly honeybees because of lack awareness of their role in pollination among farming community and adverse impact of indiscriminate use of more toxic pesticides and changing climate scenario in the country. The reasons of pollinators decline are excessive use of pesticides^8–10^ disposable plastic cups^11,12^, destruction of natural habitation^13^ and intensive farming practices, mono-cropping and higher temperatures associated with climate change^14^.

Dill (*Anethum* *graveolens* L.) is an important aromatic as well as medicinal annual herb from the Apiaceae family. Seeds and leaves of this plant are used as the main edible parts^15^ and it is a widely used spice due to its pleasant spicy aroma and plenty of nutritional and medicinal properties^16–18^. Dill essential oil contains dill apiole, carvone, carvacrol, dihydrocarvone, limonene, p-cymenand α-phellandrene^15^. It is used in the treatment of several ailments viz., gripe water to relieve colic pain in babies and flatulence in young children^19^, while the seed is carminative, mildly diuretic, galactogogue stimulant and stomachic^20^. It is also used in the cure of certain urinary complaints, piles and mental disorders^21^. In India during 2019–2020, dill was cultivated in an area of 32.79 thousand ha with a production of 34.56 thousand tonnes seed and a productivity of 1054 kg/ha^22^. *Anethum* *graveolens* is a cross-pollinated crop, with small yellow flowers, diploid (2n = 22) and strongly protandrous. The inflorescence is a compound umbel, 4–16 cm in diameter and flowers bloom in a strict sequence. The main umbel is the first to bloom followed by different range umbels in order of their range^23^. Flowering is normally completed in 9–12 days and anthers dehisce in the morning and remain receptive until midday depending upon ambient temperature^24^. Flowers are homogamous and hermaphrodite. In the umbel, some flowers are entirely pistillate and some staminate, and a few are hermaphrodite. Primary umbels bear hermaphrodite flowers, whereas, the secondary and tertiary umbels bear hermaphrodite ones on the margins and staminate types in the centre^25^. The ovary of the pistillate flower contains two ovals. Yellow staminate flowers have five stamens which arise between the petals. The staminate flowers contain noticeable quantity of nectar and have a strong odour^25^, attracting mainly bees, flies, and other pollinators. Honeybees, particularly *Apis* *florea* have been documented as the most dominant floral visitors of dill flowers^26^.

These tiny flowers make small umbellate flower bunch which provides a right landing platform for pollinators. The relative significance of insect pollinators for reproductive success of any cross pollinated plant species depends on availability of pollen and nectar, visitation frequency and ability to deposit pollen on the stigma in single visit^27,28^. The census of pollinator’s community of dill is unknown for semi-arid region of Rajasthan, India so far. Earlier studies reported honeybees, solitary bees, syrphids, muscids, some beetles and butterflies species as floral visitors of seed spices^26,29,30^. However, many researchers have reported in their study that the most common floral visitors need not always be the most effective pollinators of a particular crop^31–34^. Effective pollination is determined by pollinator’s visitation frequency, foraging rate and transfer of viable pollens to flower’s receptive stigma. Literature also supports the correlation of pollinator’s role with quantitative and qualitative yield attributes i.e. number of higher seed set/umbel, more uniform seed maturation, higher yield, seed size and test weight, oil content in seed, and increased seed germination in cross pollinated crops^35,36^. In dill, Warakomska et al^37^ reported that the isolation of flowers from pollinators decreased seed yield by 30% in 1977 and 43% in 1978. In the context of above findings, the main objective of this study was to find out the diversity of different floral visitors, dynamics of foraging activity and abundance of potential pollinators of dill in semi-arid region of India, for future conservation. Attempts were also made to find out the impact of different modes of pollination mainly bee pollination with honeybee *Apis* *mellifera,* on yield and quality of dill seed.

## Materials and methods

### Study area

The present investigation was carried out at the Research Farm of ICAR-National Research Centre on Seed Spices, Ajmer, Rajasthan, India, considered under semi-arid region of the country. Field trials were conducted during winter cropping season (November to March) of 2016–2017 and 2017–2018. The study site is surrounded by Aravalli hills, located at the coordinates 26°27′0″ N latitude, 74°38′0″ E longitude, with 460 m msl altitude^38^. This region is exposed to extremes of weather, where temperatures were ranged between 37 and 48 °C and 6 to 12 °C, during summer and winter, respectively. An annual rainfall of 300–550 mm, 60–90% relative humidity and medium to heavy fog were also observed during study period. Meteorological data of experimental location for study period during both the years are given in Table 1. The surrounding area of 1.5 km is dominated with a variety of wild trees, many shrubs and flowering weeds provided a good-natural habitat and forage to honeybee species round the year, as it contained many hives of *Apis* *florea* and *Apis* *dorsata*. The other pollinators i.e. *Halictus* sp., *Musca* sp., *Syrphid* flies etc. were also available in the natural habitation at experimental vicinity. Honeybees of *A.* *mellifera* species were visited on flowers of dill seeds from the bee hives positioned 200 m from experimental field.

**Table 1:** Weekly weather data of study period during cropping season of the years 2016–2017 to 2017–2018.

Meteorological week	2016–2017					2017–2018				
	Temperature °C		Relative humidity (%)		Rainfall (mm)	Temperature °C		Relative humidity (%)		Rainfall (mm)
	Min	Max	Morning 7.40 h	Day time 14.40 h		Min	Max	Morning 7.40 h	Day time 14.40 h	
40	23.3	32.6	92.3	69.4	136.8	19.7	36.4	82.3	47.3	0.0
41	22.4	32.8	90.7	59.3	0.0	16.9	37.4	73.7	34.3	0.0
42	19.9	33.6	80.6	59.3	0.0	15.4	36.6	69.7	30.0	0.0
43	17.9	33.1	82.9	40.6	0.0	12.4	34.6	70.0	31.6	0.0
44	12.3	32.0	88.9	30.9	0.0	12.7	33.0	54.7	44.6	0.0
45	10.1	31.4	90.7	36.0	0.0	11.4	31.4	72.1	46.3	0.0
46	9.4	29.7	93.6	41.7	0.0	11.9	26.7	75.4	59.4	0.0
47	8.0	30.4	92.1	39.7	0.0	5.6	27.9	76.6	48.3	0.0
48	8.3	30.4	92.3	42.6	0.0	7.1	27.6	75.4	51.9	0.0
49	7.7	28.6	93.1	44.3	0.0	8.0	24.9	69.9	61.1	0.0
50	10.9	28.1	93.6	48.0	0.0	7.6	21.6	81.0	77.7	7.2
51	6.3	27.4	92.6	57.1	0.0	5.0	26.4	89.3	72.0	0.0
52	7.0	26.4	92.0	49.7	0.0	5.0	25.4	73.4	70.6	0.0
1	8.0	23.4	93.3	60.9	0.0	3.1	23.6	85.3	62.1	0.0
2	3.1	19.4	91.6	58.9	0.0	3.9	25.0	83.9	61.3	0.0
3	5.4	21.9	90.6	45.6	0.0	4.9	26.9	88.4	46.4	0.0
4	10.9	23.4	93.7	64.6	24.3	4.4	24.9	87.9	59.4	0.0
5	8.8	24.9	93.4	69.9	0.0	7.1	26.6	81.6	50.6	0.0
6	4.9	23.1	91.9	54.7	0.0	7.0	25.3	85.3	50.6	0.0
7	8.1	29.4	91.1	46.9	0.0	6.9	28.2	85.3	44.9	0.0
8	7.1	28.1	91.7	51.1	0.0	13.0	31.4	80.3	46.4	0.0
9	10.1	30.5	91.3	42.9	0.0	12.9	32.6	81.7	37.9	0.0
10	10.1	27.4	90.0	54.9	0.0	11.6	32.4	75.9	39.6	0.0
11	11.6	31.3	91.9	37.9	0.0	14.9	33.8	81.9	55.8	0.0
12	16.4	36.3	91.0	36.4	0.0	15.5	32.3	81.1	52.4	0.0
13	21.3	37.4	91.3	36.6	0.0	14.9	37.8	71.4	39.9	0.0
14	16.0	36.1	83.3	28.0	0.0	22.2	39.2	73.0	32.6	0.0
15	21.9	41.4	87.6	40.0	0.0	21.4	38.0	86.1	35.4	0.0
16	25.7	38.8	85.1	42.4	0.0	22.3	38.3	73.6	26.9	0.0
17	22.0	38.1	91.0	44.4	0.0	24.1	40.9	64.7	31.3	0.0

## Experiment design

**Figure 1:**
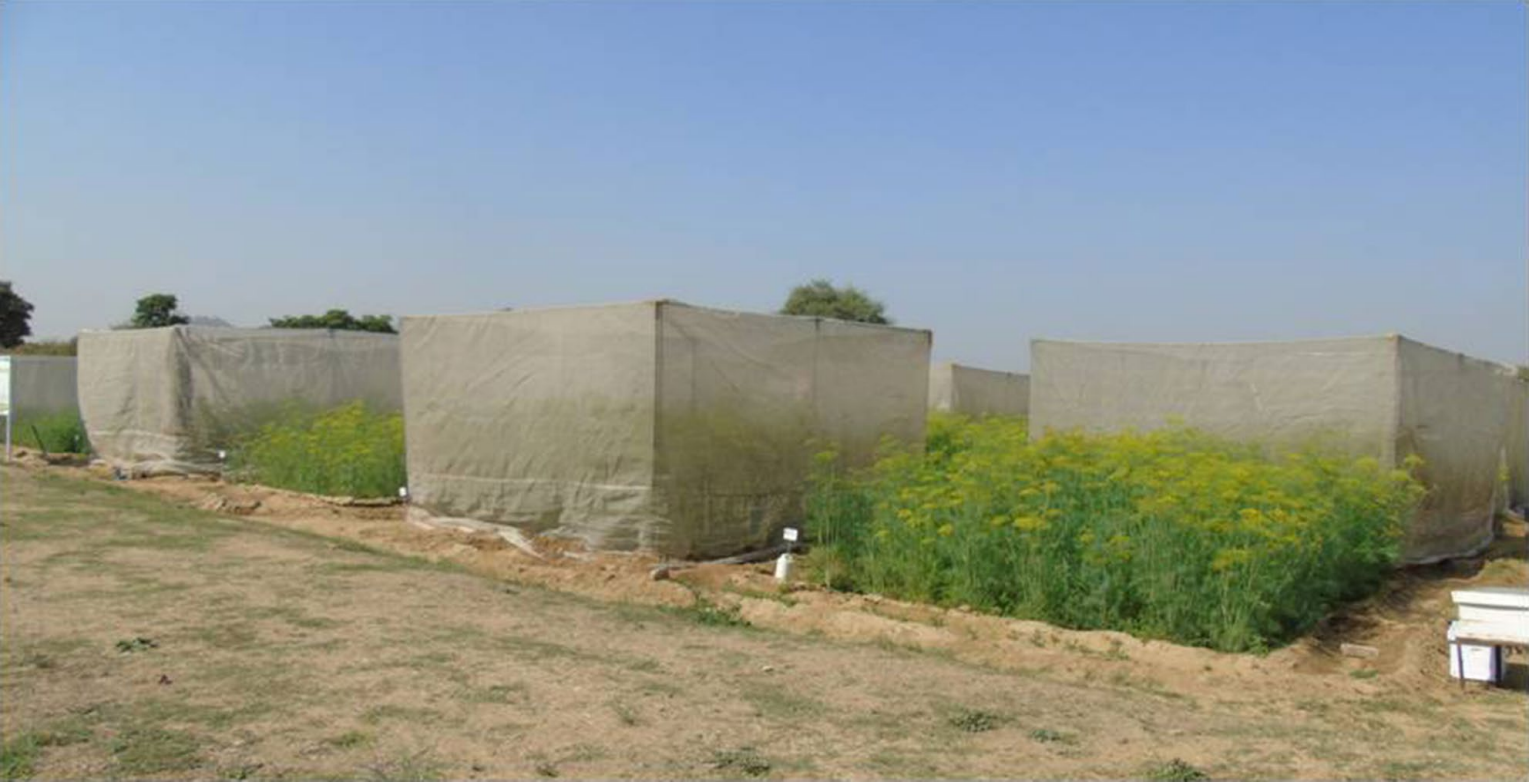
Design of field experiment showing insect cages for pollination study in dill seed crop.

The field experiment was designed to study the diversity of different floral visitors, dynamics of foraging activity, abundance of potential pollinators and effect of different mode of pollination on yield and quality of dill seeds in semi-arid region of the country. The field trials were laid out in a randomized block design with five treatments, replicated four times (Fig. 1). Dill seeds of variety AD-1 were sown in individual plots of 20 m^2^ (5 × 4 m) under 50 × 30 cm crop geometry (row to row and plant to plant spacing) following recommended package of practices for dill seeds^39^. The five treatments as independent variables namely T_1_-without insect pollination (WIP-caged) as control, T_2_-open pollination (OP), T_3_-bee pollination (BP-caged), T_4_-open pollination with jaggery solution 10% and T_5_-open pollination with sugar solution 10% were applied as different modes of pollination. Jaggery and sugar solution were sprayed on the flower canopy of dill seeds at 20–30% flowering initiation stage to attract bees and other floral visitors for pollination. Plants of two plots (WIP and BP) in each replication were covered by insect cages made of GI pipes, lined with 16-mesh insect proof nylon nets. The insect cages measuring of 5 × 4 × 3.5 m (length × width × height) were installed on plants before initiation of flowering^40^ to kept plants free from the activities of any insect species within the insect cage of control plots (WIP) in each replication throughout the flowering period. At the same time, a four-framed colony of *A.* *mellifera* was kept inside the cages of bee pollination plots (BP) in all replications to ensure pollination services, while the plants in open pollination plots were exposed to natural pollination by all floral visitors. It is confirmed that all methods were performed in accordance with the relevant guidelines/regulations/legislation.

### Floral visitor diversity

For this study, data on floral visitors’ diversity and their population effectiveness were recorded by sampling methods of direct observation and sweep net collection^41–44^. Observations on floral visitor diversity consisted sweep-netting the floral visitors throughout the blooming period^40^ i.e. from the first week of January to the last week of March. The sweep net catches were recorded each time in open pollination plots in all the four replicates, where no plant protection measures were taken throughout the cropping period. In each observation, 4 to 5 sweep-net catches were made for 10 min on 20 square meters of flowering area of the entire plot/replication. These observations were made five times at 09.00, 10.00, 12.00, 14.00 and 16.00 h at weekly intervals throughout the flowering period. Floral visitors were also recorded at same time interval by visual counting from 1 m^2^ flower canopy to determine the mean abundance/m^2^ per day and proportion (%) of individuals from total floral visitors. The observation timing was delayed till 09.00 h during the period due to heavy fog deposited on plant canopy and there was lack of bright sunshine. Specimens of floral visitors were collected, preserved and identified based on available literature information, but all specimens are not assigned to species level. The identifications of the insects were confirmed by the taxonomist.

### Population dynamics

The insect species visited flowers and transport pollen grains in the body were considered as pollinator of dill seeds. Population dynamics study of selected floral visitors was conducted to know the foraging activity and temporal abundance on dill seeds flowers. Foraging activity of 14 different floral visitors was recorded as population fluctuation (number of floral visitors visited/m^2^ bloom area per five minutes) at weekly interval throughout the flowering period. Observations of pollinator’s flower visitation were conducted by scan sampling methods^45^. The data on number of floral visitors/m^2^ bloom area per five minutes was recorded four times (10.00, 12.00, 14.00 and 16.00 h) per day at weekly interval and then converted to a mean population. Similarly, the temporal abundance of 10 most important and frequent floral visitors was studied on dill flowers. Abundance of flower visitors was studied as the number of floral visitors/m^2^ boom canopy per minute to determine their foraging rates during particular period of the day. Observations on number of floral visitors/ m^2^ boom canopy per minute were recorded at hourly intervals from 06.00 to 10.00 h and two hourly intervals from 10.00 to 18.00 h for 10 calm and clear sunny days^43^ during peak flowering stage.

The foraging rate of 3 honeybee species (*A.* *mellifera,* *A.* *florea* and *A.* *dorsata*) was also studied as the number of flower umbels visited per minute^46^ as well as the number of plants visited per five minutes, where the observations were recorded six times a day (08.00, 10.00, 12.00, 14.00, 16.00 and 18.00 h) for 10 calm and clear sunny days to determine their potentiality within species as pollinator of dill seeds for semi-arid region of the country.

### Pollination effectiveness

Pollination effectiveness was measured in term of per unit area yield appreciation, one thousand seed test weight and essential oil contents. The five different treatments (modes of pollination) as described in the sub-heading experimental design were followed in all four replications to determine the impact on seed yield^47^, test weight and essential oil content. Two open plots (T_4_ and T_5_) per replicate were sprayed with jaggery solution 10% and sugar solution 10% as indigenous bee attractants at 20% flowering stage^48,49^ to increase *Apis* pollinators activity up to 3rd day of treatment. Data on yield parameters were recorded on randomly selected five plants /plot and on the total number plants harvested separately for each mode of pollination. For the assessment of quality of dill seeds in terms of test weight and essential oil contents, 1000 clean seeds were randomly selected using seed counter machine and test weight was measured using physical balance. The essential oil was extracted from 30 g dried dill seed using hydro-distillation method^50^.

### Data analysis

The statistical analysis was performed using the SPSS statistical software. We calculated mean population of each floral visitor per day for entire flowering period and their per cent proportion of total floral visitors to study the diversity of insect pollinators of dill seeds. Weekly population fluctuation and abundance of potential and frequent floral visitors on dill flowers were also studied by calculating means, SE (m) and p value by ANOVA analysis using statistical software OPSTAT developed in Haryana Agricultural University, Hisar, India. The data on population fluctuation (number of floral visitors visited/m^2^ bloom area per five minutes), and temporal abundance (number of floral visitors per square meter bloom area /minute) were recorded separately for two years and then converted into pooled analysis to represent the population dynamics. Similarly, seed yield per plot were recorded separately for two consecutive years and converted into kg per hectare yield, test weight per 1000 seeds and essential oil content in percentage. After conversion into appropriate unit, data of both the years for each parameter were pooled and mean values for yield and seed quality (test weight of randomly selected 1000 seeds and essential oil content) were subjected to statistical analysis using analysis of variance (ANOVA). Means were compared by use of Duncan’s Multiple Range test (DMRT) at P ≤ 0.05.

### Confirmation of experimental research

The field experimental research and collection of plant material was carried out with compliance of our institutional guidelines, hence it is confirmed that all methods were performed in accordance with the relevant guidelines/regulations/legislation.

## Results

**Figure 2:**
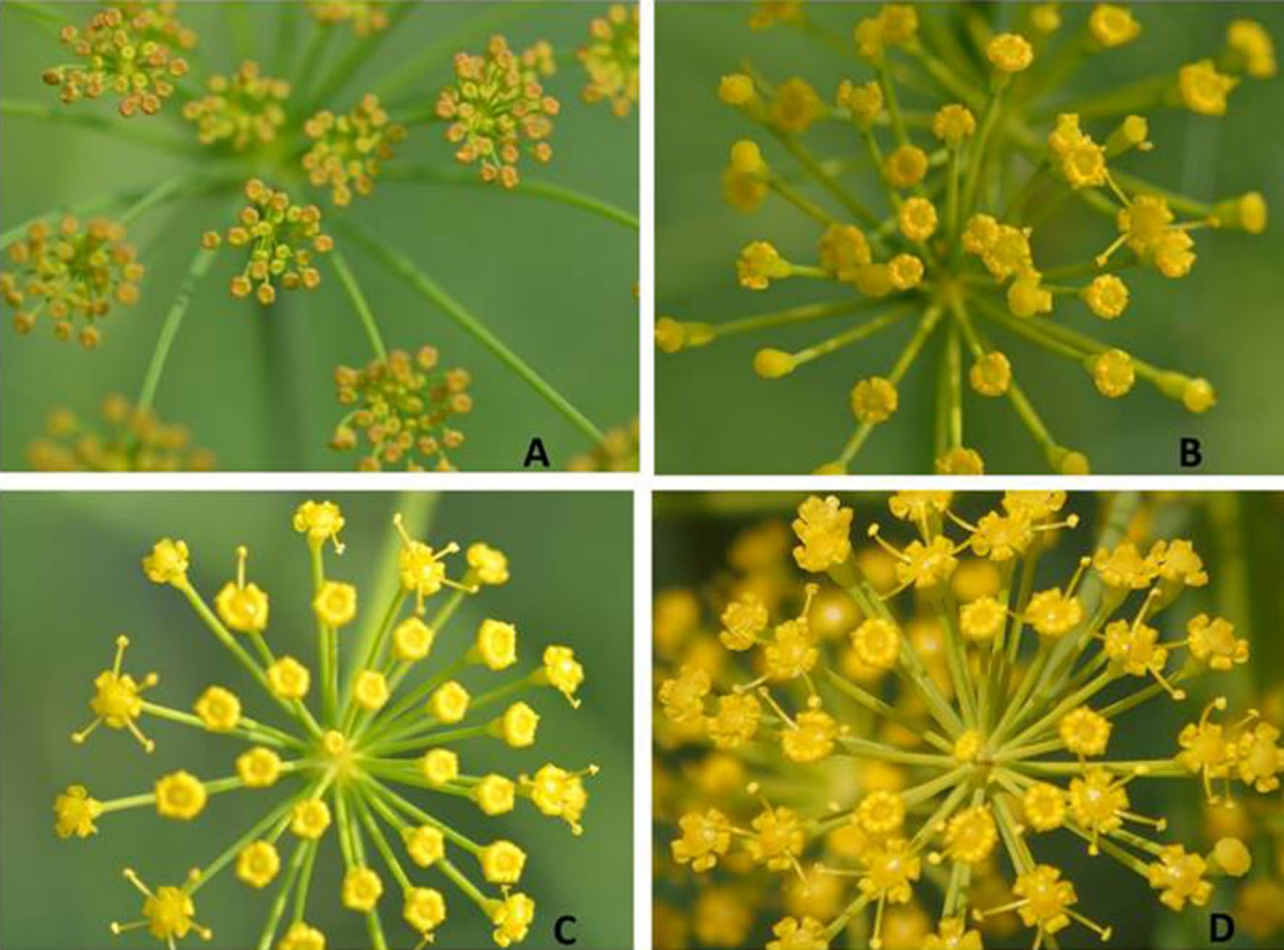
Flower biology of dill seeds (**A **flower bud stage; **B** flower bud opening stage; **C** partially opened flower, **D** fully opened flower).

The results of present study conducted on diversity of floral visitors, dynamics of foraging activity, abundance of potential pollinators and effect of different mode of pollination on yield and quality of dill seeds are described in this section and data presented in tables. *Anethum* *graveolens* is a winter season crop, sown in the month of October and plants initiate flowering in 70–90 days depends on existing climatic conditions. The flowering of dill seeds lasted for about 2 months in a strict sequence starting from main umbel to different range umbels. Dill seeds produce small yellow flowers, inflorescence is a compound umbel, 4–16 cm in diameter and are strongly protandrous. Flowers start opening in the morning between 8.30 and 9.30 h, and fully opened between 12.00 and 14.00 h. A single dill seeds flower lasted for 9–12 days consider its longevity (Fig. 2). Anthers dehisce in the morning and remain receptive throughout the day till 16.00 h. A number of insects visit dill flowers throughout the day to collect nectar and pollen as flower resources are offered to visitors and visited flowers get pollinated.

### Floral visitor diversity

The floral visitors of *A.* *graveolens* L. composed of 28 insect species belonging to 14 families of 6 orders. Among the various floral visitors on dill flowers, the majority belonged to Hymenoptera (10 species) followed by Diptera with eight species, while three species each were from Hemiptera, Coleoptera and Lepidoptera and one species was from Neuroptera order (Table 2). The hymenopteran pollinators included honeybee species *Apis* *florea,* *A.* *dorsata,* *A.* *mellifera*, a solitary bee, *Halictus* sp., and one unidentified species. The highest mean population was recorded for *A.* *florea* (62.6 bees/m^2^ per day) followed by *A.* *mellifera* (32.6 bees), *Apis* *dorsata* (19.6 bees), and *Halictus* sp. (9.81 bees), on dill flower ecosystem. The other Hymenoptera species recorded were *Polistes* *hebraeus,* *Ceratina* *sexmaculata* Smith*,* *Xylocopa* sp., *Camponotus* sp. and unidentified hymenoptera sp 1, but their numbers were negligible. *A.* *florea* and *A.* *mellifera* were the most frequent and dominant floral visitors constituting 43.69 and 22.76% proportion of total Hymenoptera and 30.14 and 15.71% proportion of total floral visitors, respectively. The second most dominant group of dill floral visitors was eight species from Syrphidae and Muscidae families of Diptera. Out of these, *Episyrphus* *balteatus* (DeGeer) and *Episyrphus* sp. (Syrphidae) and *Musca* *domestica* (Muscidae) were only recorded as the part of the pollinator’s community and the most abundant with mean population of 12.96, 6.33, and 6.09 flies /m^2^ per day, respectively (Table 2). The floral visitors of Diptera contributed 18.34% proportion of the total visitors on dill flowers for two cumulative years during winters and the proportion of flies ranged from 0.2 to 6.24% of the total visitors. The other occasional floral visitors were *Coccinella* *septempunctata* L.*,* *Menochilus* *sexmaculatus* Fab.*,* *Dysdercus* *koenighii* F. and *Oxycaranus* *laetus* (Kirby) and *Raphidopalpa* *foveicollis* Lucas (Coleoptera), *Bagrada* *hilaris* Burm. (Hemiptera), *Chrysoperla* *zastrowi* *sillemi* (Esben-Petersen) (Neuroptera), *Helicoverpa* *armigera* Hub., *Pieris* *brassicae* L. and an unidentified species (Lepidoptera), however their numbers were negligible (0.3 to 1.1 insects/m^2^ per day).

**Table 2:** Diversity of floral visitors on dill (*Anethum* *graveolens* L.) during *rabi* in semi-arid region.

Name of species	Order	Family	Mean abundance/m^2^ day^−1^	Proportion (%) of total visitors
*Apis* *dorsata* Fab	Hymenoptera	Apidae	19.6	9.44
*Apis* *florea* F		Apidae	62.6	30.14
*Apis* *mellifera* L		Apidae	32.62	15.71
*Ceratina* *sexmaculata* Smith		Apidae	4.5	2.17
*Polistes* *hebraeus* (F.)		Vespidae	0.9	0.43
*Xylocopa* sp.		Apidae	1	0.48
*Camponotus* sp.		Formicidae	2.02	0.97
*Halictus* sp.		Halictidae	9.81	4.72
Unidentified hym sp. 1			3.62	1.74
Unidentified hym sp. 2			6.6	3.18
Total Hymenoptera			143.27	68.99
*Episyrphus* *balteatus* (DeGeer)	Diptera	Syrphidae	12.96	6.24
*Episyrphus* sp.		Syrphidae	6.33	3.05
*Eristalis* sp 1		Syrphidae	5.85	2.82
*Eristalis* sp 2		Syrphidae	3.2	1.54
*Musca* *domestica* (D)		Muscidae	6.09	2.93
Musca sp 1		Muscidae	0.42	0.20
Musca sp 2		Muscidae	2.81	1.35
Musca sp 3		Muscidae	0.43	0.21
Total Diptera			38.09	18.34
*Dysdercus* *koenighii* F	Hemiptera	Pyrrhocoridae	2.49	1.20
*Oxycaranus* *laetus* (Kirby)		Lygaeidae	2.6	1.25
*Bagrada* *hilaris* (Burmeister)		Pentatomidae	0.9	0.43
*Coccinella* *septempunctata* L	Coleoptera	Coccinellidae	11.23	5.41
*Menochilus* *sexmaculatus* Fab		Coccinellidae	5.38	2.59
*Raphilopalpa* *foevicollis*			0.5	0.24
*Chrysoperla* *zastrovi* *sillemi*	Neuroptera	Chrysopidae	0.3	0.14
*Helicoverpa* *armigera* Hub	Lepidoptera	Noctuidae	0.72	0.35
*Pieris* *brassicae* L		Pieridae	1.1	0.53
Lepidoptera sp (unidentified)			1.1	0.53
Total others			26.32	12.67
Grand total			207.68	**–**

The data given in table are the pooled data of 2 years research (2016–2017 & 2017–2018).

### Population dynamics

**Figure 3:**
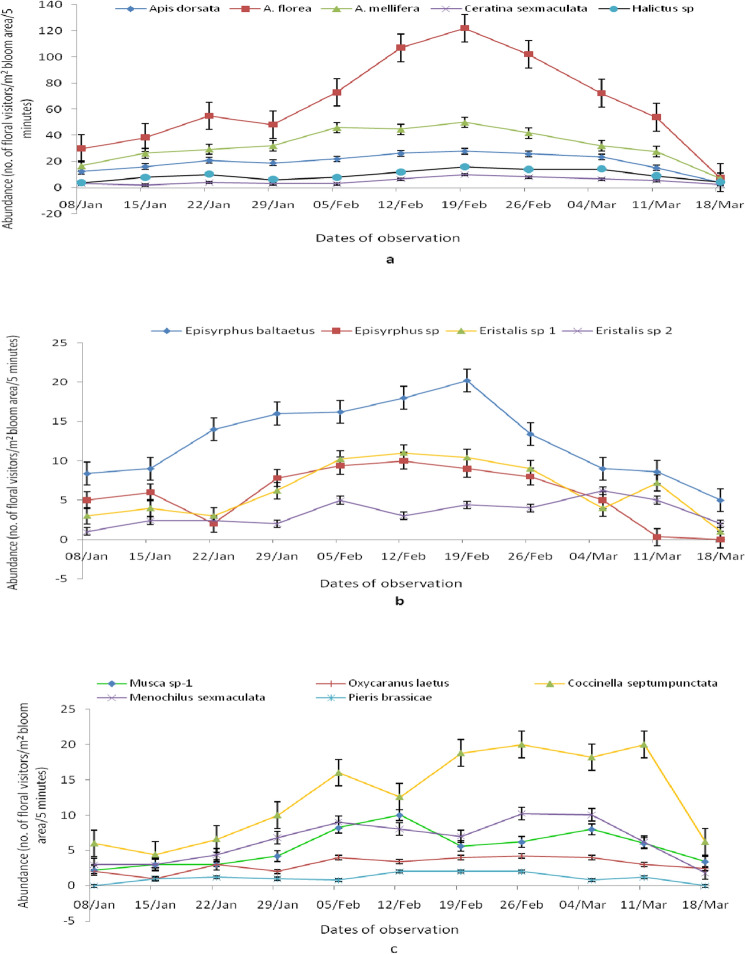
Fluctuation in populations (number of floral visitors/m^2^ bloom area per 5 min) of (**a)** bees, (**b)** Syrphids, and (**c)** other pollinators on dill seeds during flowering in semi-arid region.

The population dynamic pattern as fluctuation in population of bee floral visitors on dill seeds during entire flowering season (Fig. 3a) revealed that *A.* *florea,* *A.* *mellifera,* *A.* *dorsata* and *Halictus* sp. were the most dominant and regular pollinators in the region. The highest activities of different bee pollinators were recorded in the range of 10 to 122 bees/m^2^ bloom area/5 min with a mean of 45.2 ± 45.5 (± SD) in the third week of February. Honeybee species *A.* *florea* (122 bees/m^2^ bloom area/five minutes) were noticed as most frequent and statistically highest populated pollinator on dill seeds which was followed by *A.* *mellifera,* *A.* *dorsata*, *Halictus* sp. and *C.* *sexmaculata* (50, 28, 16 and 10 bees/m^2^ bloom area/5 min), respectively in the third week of February. Among the Syrphid pollinator, species *Episyrphus* *balteatus,* *Episyrphus* sp., *Eristalis* sp. 1 and *Eristalis* sp. 2 were recorded in the range of 6.2 to 20.2 flies/m^2^ bloom area/five minutes with different peaks and comparatively less populated regular floral visitors (Fig. 3b). During the entire flowering season, other than bees, there was uneven distribution of population dynamic pattern of all pollinators except true flies (Fig. 3c).

The diurnal dynamic pattern of most frequent and dominant floral visitors (Table 3) revealed that *A.* *florea* and *A.* *mellifera* started their activity at 08.00 h with very low numbers (0.08–0.60 bees/m^2^ bloom area/minute) and frequency. The visiting frequency increased gradually with the higher temperature and more sun light reaching peak population between 12.00 and 14.00 h showing the population in the range of 2.10 to 20.63 insects/m^2^ bloom area per minute at 14.00 h. *Apis* *florea* was the most abundant (20.63 bees/m^2^ bloom area/minute) pollinator followed by *A.* *mellifera* (15.45 bees/m^2^ bloom area/minute) and both the pollinators were statistically differed in abundance (P ≤ 0.05) with a mean value of 7.61. Among the bee species, only *A.* *dorsata* sustained its activity throughout the day, starting at 07.00 h and reaching the peak (8.05 bees/m^2^ bloom area/minute) at 12.00 h followed by a decline to negligible number (0.4 bees/m^2^ bloom area/minute) at 18.00 h. The abundance of *A.* *dorsata* on dill seeds was statistically lowered (P ≤ 0.05) to *A.* *mellifera* and *A.* *florea* but higher to remaining floral visitors at all intervals. *Halictus* sp. started their activity at 08.00 h, then steadily increased and reached to its highest population (3.6 bees/m^2^ bloom area/minute) followed by a completely checked out after 16.00 h. Syrphid species were available in the field of dill crop throughout the day, either sitting or flying on flower umbels and exhibited a population fluctuation pattern. *Episyrphus* *balteatus*, *Episyrphus* sp. 1 and *Eristalis* sp. started their activity at 07.00 h with very few numbers (0.05 to 1.00 flies/m^2^ bloom area/minute), thereafter their population fluctuations were statistically varied (P ≤ 0.05) depending on species with their peaks at different hours of the day (Table 3). The other floral visitors i.e. *Ceratina* *sexmaculata* and unidentified hymenoptera sp. 2 were also recorded on dill flowers at different hours of the day almost in similar pattern, whereas *Musca* sp. 2 was observed throughout the day from 06.00 to 18.00 h with peak population of 4.13 flies/ m^2^ bloom area per minute at 12.00 h.

**Table 3:** Temporal abundance of important floral visitors on dill crop in semi-arid region during winter cropping season.

Insect visitor	Mean population of floral visitors per 1 m^2^ boom canopy/minute during different hours									Mean
	06.00	07.00	08.00	09.00	10.00	12.00	14.00	16.00	18.00	
*Apis* *dorsata* F	0.0	0.1	0.15	2.5	4.6	8.05	7.36	2.18	0.4	2.82
*Apis* *florea* F	0.0	0.0	0.6	5.43	11.64	17.23	20.65	9.2	0.0	7.19
*Apis* *mellifera* L	0.0	0.0	0.08	4.53	8.6	13.25	15.45	5.74	0.0	5.29
*Ceratina* *sexmaculata* Smith	0.0	0.05	1.02	1.95	3.3	5.05	5.83	2.69	0.51	2.27
*Halictus* sp.	0.0	0.0	0.1	1.3	2.9	3.55	3.6	1.6	0.0	1.45
Unidentified Hymenoptera sp 2	0.0	0.0	0.0	1.55	3.05	3.8	4.45	1.6	0.01	1.61
*Episyrphus* *balteatus* (De Geer)	0.0	0.1	1.55	1.58	3.56	6.6	6.03	2.97	1.0	2.6
*Episyrphus* sp. 1	0.0	1.0	1.09	2.15	1.7	5.3	6.52	3.0	0.0	2.31
*Eristalis* sp.	0.0	0.05	0.55	2.95	1.58	3.96	4.15	2.4	0.0	1.74
*Musca* sp. 2	0.1	0.58	0.35	1.48	2.13	4.13	2.1	1.55	1.1	1.5
Mean	0.01	0.19	0.55	2.54	4.31	7.09	7.61	3.29	0.30	2.88
SE (m) ±	0.01	0.02	0.07	0.08	0.10	0.32	0.46	0.30	0.01	–
CD (p = 0.05)	0.03	0.06	0.22	0.23	0.30	0.97	1.38	0.91	0.04	–

The data given in table are the pooled data of 2 years research (2016–17 & 2017–18).

*√x + 1 transformed value of tabulated data were used for statistical analysis.

**Figure 4:**
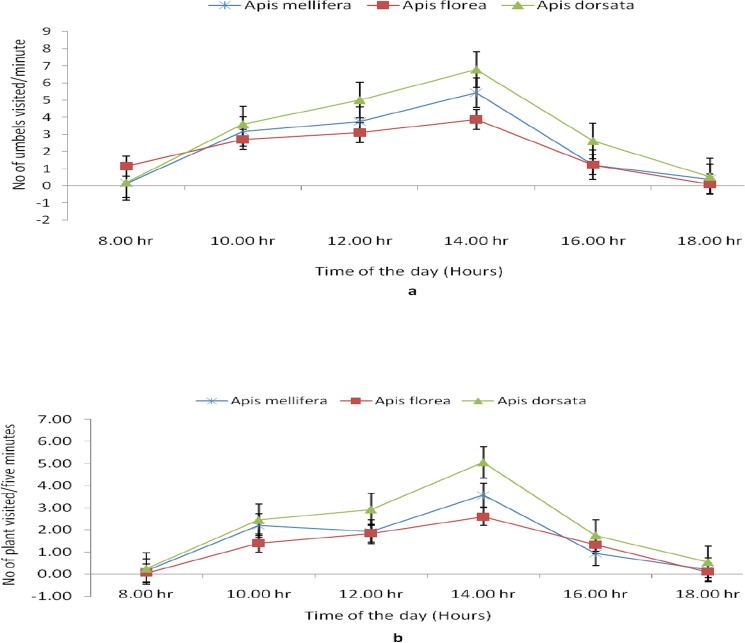
Foraging rates of honeybee pollinators on dill seeds flowers (**a)** number of umbels visited per minute, (**b)** no of plant visited/five minutes during full bloom in February under semi-arid region.

The foraging rate of three honeybee species on dill seeds flowers as number of flower umbels visited per minute revealed that their foraging visits were noticed between 08.00 and 09.00 h and continued throughout the day. The foraging rate of *A.* *dorsata* was the highest (visiting frequency: 6.78 flower umbels visited per minute) followed by *A.* *mellifera* and *A.* *florea* which visited 5.43 and 3.88 flower umbels per minute, respectively at 14.00 h. Thereafter, there was a reduction in flower visiting frequency of honeybee pollinators (Fig. 4a). *A.* *dorsata* also visited the maximum number of dill plants (average 5.05 plants per five minutes) followed by *A.* *mellifera* (3.58) and *A.* *florea* (2.60) during 14.00 h (Fig. 4b).

### Pollination effectiveness

The effect of different modes of pollination on seed yield and quality attributes of *A.* *graveolens* was studied under statistically well designed field experimentation and data presented in Table 4, revealed that, the insect pollinators significantly affected the seed yield (P ≤ 0.05, Duncan’s Multiple Range test DMRT). The effect of different modes of pollination on yield was evident as the lowest seed yield of 958.7 kg/ha was recorded in the plots considered as control (WIP) having no input of floral visitors, and significantly inferior to all adopted modes of pollination. The highest seed yield of 1505.63 kg/ha was recorded in open pollination with jaggery solution 10% treated plots at par with 1459.48 kg/ha seed yield obtained in open pollination (OP) plots and 1432.5 kg/ha seed yield recorded in plots pollinated only with *A.* *mellifera* (BP) honeybee species under cage condition (P ≤ 0.05, Duncan’s Multiple Range test DMRT). Among the four modes of pollination, significantly lowest seed yield of 1317.5 kg/ha was recorded in sugar solution 10% treated plots. Overall, the four modes of pollination had a significant impact on seed yield showing 37.4 to 57.04 percent increase over control. Similarly, the quality traits (test weight and essential oil %) of dill seeds were also influenced by different modes of pollination. All pollination treatments were statistically at par (3.16 to 3.34 g) for test weight within treatment but significantly better to control (P ≤ 0.05, Duncan’s Multiple Range test DMRT). Insect pollination also increased the essential oil having carvone and dill apiole content from 1.71% in control to 2.16–2.19% in the treatments but statistically at par with each other (P ≤ 0.05, Duncan’s Multiple Range test DMRT), respectively (Table 4).

**Table 4:** Effect of different modes of pollination on yield and quality of dill seeds in semi-arid region during winter cropping season.

Treatment	Seed yield (kg/ha)	Per cent change compared to			Test weight per 1000 seeds (g)	Essential oil (%)
		WIP	OP	BP		
T_1_-WIP-caged	958.7^d^	–	− 52.2	− 49.4	1.70^b^	1.71^b^
T_2_-OP	1459.48^ab^	52.2	–	− 1.8	3.21^a^	2.16^a^
T_3_-BP-caged	1432.5^b^	49.4	− 1.8	–	3.24^a^	2.19^a^
T_4_-Jaggery solution 10%	1505.63^a^	57.0	3.1	5.1	3.34^a^	2.17^a^
T_5_-Sugar solution 10%	1317.5^c^	37.4	− 9.7	− 8.0	3.16^a^	2.06^a^

The data given in the table are pooled of 2 years research (2016–17 & 2017–18); values are average of 4 replications.

*WIP* without insect pollination, *OP* open pollination, *BP* bee pollination is not all bees pollination but just honeybee pollination.

Averages followed by the common letters in a column are not statistically different at (P ≤ 0.05) according to Duncan’s multiple range test (DMRT).

## Discussion

Typically small, actinomorphic yellow flowers of dill seeds produce plant resources like nectar and pollen to attract wide range of insects, bees and wasp^17^. Small individual flowers make umbellate which develop into a flower bunch known as umbels^51^, they provide an excellent landing platform for pollinators. In the present study, flowers of dill seeds start opening in the morning between 8.30 to 9.30 h, and fully opened between 12.00 to 14.00 h. A single dill seeds flower lasted for 9 to 12 days confirm as flower longevity in winter under semi-arid conditions. Anthers dehisce in the morning and remain receptive throughout the day. Dill seeds flowers are also open during night but no floral visitors were reported due to heavy fog during study period. A number of insect species visit dill flowers throughout the day to collect nectar and pollen as flower resources are offered to visitors and as resulted flowers get pollinated. In the study, twenty eight species of floral visitors from fourteen families of six orders were recorded for foraging dill crop in semi-arid region of Rajasthan, India throughout flowering in winter cropping season (January-March). In the study area, *Apis* *florea* was recognized as the most frequent and dominant pollinator of dill followed by *A.* *mellifera* and *A.* *dorsata*. Earlier, Chaudhary and Singh^47^ reported that *A.* *florea* was the prominent pollinating agent in different seed spice crops under arid to semi-arid regions corroborates to the present study. The honeybee species *Apis* *mellifera,* *A.* *dorsata* and *A.* *florea* were also reported as major and potential pollinators of coriander^52,53^, fennel^40,54^, cumin^38^ and black cumin^55,56^. Abundance and composition of pollinators vary with the varied geographical area, latitude and the time^57^. In our study, the other pollinators recorded to visit dill flowers were true flies *Episyrphus* *balteatus*, *Episyrphus* sp., and *Eristalis* sp. 1 and *Eristalis* sp. 2 from Syrphidae and four species from Muscidae. Previous studies have reported syrphid flies to be good pollinators of many crops such as coriander^47^, fennel^58^ and many other seed spice crops^59^. In this study, more than two-third of the total floral visitors in dill crop ecosystem belonged to Hymenoptera and Diptera and hence referred to as majority pollinating insects for seed spices.

The flowers of dill seeds received floral visitors in first week of January with lower numbers in our study due to flowering initiated on crop at this stage. The higher populations of the majority of floral visitors were noticed between second and third weeks of February due to peak of flowering and increased availability of nectar and pollen. The peak activities of these pollinators varied among species due to their foraging nature and availability of nectar and pollen in the flowers. No literature available on this aspect particularly on dill seeds, hence, it could not be discussed in length.

The activities of most of the floral visitors on dill seeds flowers were initiated at 8.00 h and peak activity of most of the pollinators were observed from 12.00 to 14.00 h in our study, may be due to the availability of nectar and pollen was highest during this period. Koul^53^ observed that peak activity during this period was also due to visual impact of the compound umbel along with presence of exposed nectar and availability of pollen. In diurnal dynamic pattern, the hymenopterans, *A.* *florea*, *A.* *mellifera* and *Halictus* sp. were more abundant at 14.00 h, as most of the bees consume nectar and its availability increased after 12.00 h. However, wild honeybee *A.* *dorsata* visited dill flowers throughout the day with higher numbers at 12.00 h. Kapil et al.^60^ and Bhalla et al.^61^ also reported similar findings on different crops including seed spices. Foraging activities of other floral visitors in the present study exhibited an indefinite pattern. *Episyrphus* *balteatus*, *Episyrphus* sp and *Eristalis* sp 2 foraged in sufficient numbers for longest period till 17.00 h, while none of floral visitors except *Musca* sp. 2 was observed foraging at 06.00 h on *A.* *graveolens* flowers in winters under semi-arid region of Rajasthan, India. Continuous foraging activity of *Musca* sp. was reported on coriander from morning till evening (06.00 h–18.00 h) during winter^47^, whereas Sikdar et al.^59^ noticed the higher activity of dipteran flower visitors during the morning (09.00 h–10.00 h).

Foraging rate and flower visitation frequency of insect pollinators on cross pollinated crops are important to judge the pollination efficiency of different species. Earlier researchers also reported the dependency of floral visitors foraging rates and visitation frequency on various factors including quantity of floral rewards^62^, sugar concentration in nectar^63^, length of proboscis and instinctive foraging dynamics^64^ and some abiotic factors i.e., wind velocity, ambient temperature, relative humidity and intensity of light^65^. In this study, the highest visitation frequency on dill flowers was recorded for *A.* *dorsata* while *A.* *florea* had low visitation rate and greater stay time with highest pollen deposition because of small size and better foraging habit for seed spices. The higher foraging rate of pollinators enhanced the pollen rubbing of flowers and simultaneously increased the seed set and seed maturity rate in dill. Singh et al.^66^ reported that higher foraging rate and visitation frequency created higher chance of pollination, whereas Engel and Irwin^67^ observed that there was no standard rule and the insects with high visitation rates could be poor pollen depositors. In another study, *A.* *florea* was rated as lower forager but a more successful pollinator for different seed spices^26^.

The seed yield and quality of dill seeds (*A.* *graveolens*) were influenced significantly by the managed pollination services through various floral visitors. Sihag^68^ found that insect pollinators significantly enhanced the yield and quality of seeds in cross pollinated cruciferous and umbelliferous plants. In the present study, 57 per cent higher yield of dill seed was obtained in open pollination with 10% jaggery solution spray treatment over control due to additional input of other pollinators allowed from natural sources during entire flowering season. Bee pollination with *A.* *mellifera* under insect cage condition also appreciated the seed yield of dill seeds by 49.4% over control plots shows importance of bees alone for pollination in this seed spice crop. Meena et al.^38^ found in their earlier study that bee pollination increased the yield of cumin by 40.03%. The number of seeds set per plant in dill was significantly greater in open plots than control (caged plots). Verma and Dwivedi^69^ also concluded that insects visiting the flower enhance the rate of fruit setting by promoting cross pollination. Pollination management in various cross pollinated crops through honeybees and true flies, resulted in higher fruit set, seed set, test weight and oil content^28,47,68,70,71^ and these reports support the findings of the present research. In our study, jaggery and sugar solution 10% used as indigenous bee attractants, where jaggery solution 10% increased the seed yield significantly as compared to other treatments but at par with open pollination. Spraying of 10% jaggery solution enhanced the activity of *A.* *dorsata,* *A.* *florea* and *A.* *cerana* on *Allium* *cepa*^48^ and *A.* *cerana* on buckwheat^49^. The impact of managed pollination on improvement of the seed quality parameters in dill over control conforms to the findings of Sihag^68^ that pollinators greatly influence seed size, test weight (weight/1000 seeds) and seed germination percentage in cruciferous crops. Proper pollination can improve the quantity and quality of fruits, nuts, oils, and other crops produced^72^. Meena et al.^38^ also recorded higher essential oil content in cumin seeds of bee pollinated plants than control plots.

## Conclusion

The investigation indicated that a total of twenty eight insect species visit the flowers of dill seeds crop throughout the flowering season in semi-arid regions of the country. Favourable time for foraging activities of most of the floral visitors is between 12.00 and 14.00 h. It is concluded from the present study that the honeybees for sure are good pollinators for the crop, and hence, there is need to conserve by enriching their habitat. We also recommend the need-based use of safer insecticides for the control of pest insect like aphid prior to flowering which is least disruptive to pollinators in the dill seed crop. If pollinator’s performance is poor, even during peak of flowering, a spray of 10% jaggery solution can be recommended to dill producers to attract pollinators. Studies on further quantification of their impacts and ways to enhance their utility in pollination management will help increasing dill production and productivity in semi-arid regions.

## Data Availability

The datasets generated and/or analysed during the current study are not publicly available due “The year wise data of the concluded study were submitted to the PME Cell (Priority setting monitoring and evaluation cell) of the institute in the form of annual progress report of the project after discussion in Institute Research Advisory Committee Meeting” but are available from the corresponding author on reasonable request.
